# Application of Intelligent Recommendation Techniques for Consumers' Food Choices in Restaurants

**DOI:** 10.3389/fpsyt.2018.00415

**Published:** 2018-09-04

**Authors:** Xinke Li, Wenyan Jia, Zhaofang Yang, Yuecheng Li, Ding Yuan, Hong Zhang, Mingui Sun

**Affiliations:** ^1^College of Communication Engineering, Chongqing University, Chongqing, China; ^2^Department of Neurosurgery, University of Pittsburgh, Pittsburgh, PA, United States; ^3^College of Computer & Information Science, Southwest University, Chongqing, China; ^4^Image Processing Center, School of Astronautics, Beihang University, Beijing, China; ^5^Department of Electrical and Computer Engineering, University of Pittsburgh, Pittsburgh, PA, United States

**Keywords:** food choice, consumer preference, eating behaviors, food's attributes, matrix tri-factorization, food recommendation system, robotic restaurant, non-human waiter/waitress

## Abstract

Currently, there has been a new trend in applying modern robotics, information technology, and artificial intelligence to restaurants for improvements of food service, cost-effectiveness, and customer satisfaction. As robots replace humans to serve food, there is a clear need for robotic servers to help consumers select foods from a menu that satisfies their preferences such as taste and nutrition. However, currently, little is known about how eating behaviors drive food choices, and it is often difficult for consumers to make choices from a variety of foods offered by the typical restaurant, even with the assistance from a human server. In this paper, we conduct an exploratory study on an intelligent food choice method that recommends dishes by predicting individual's dietary preference, including ingredients, types of spices, price, etc. A multi-attribute relation matrix tri-factorization (MARMTF) technique is developed for a relation-driven food recommendation system. First, the user's ordering history and their rating scores of the foods in the menu are gathered and represented by a user-dish rating matrix. Next, the attribute relations of the ingredients, spicy level, and price of each food choice are extracted to construct a group of the relation matrices. Then, these matrices are integrated into a large block matrix. In the next step, a matrix tri-factorization algorithm is employed to decompose the block matrix and fuse the complex relationships into matrix factors. Further, a set of approximation block matrices are constructed and the predicted food rating matrix is generated. Finally, the foods (dishes) with sufficiently high preference scores are recommended to the consumers. Our experiments demonstrate that the MARMTF technique can provide effective dish recommendation for customers. Our system significantly simplifies the daunting task of making food choices and has a great potential in providing intelligent and professionally trained non-human waiters and waitresses for employment by future restaurants.

## Introduction

In recent years, there has been an interesting new trend to apply modern robotics, information technology and artificial intelligence (AI) to restaurants. Tablet computers for food ordering have been widely utilized in many countries (1–3). Robotic restaurants without human waiters and waitresses have been in operation, such as those in Canada (4), Japan (5), and Singapore (6). Although these trends have great potential in improving restaurant service, reducing cost, and enhancing customer satisfaction, the reduction or elimination of human interaction with customers on food choices significantly increases the problem in selecting a dish from a long restaurant menu. Although food flavor and appearance have been important features for consumers choosing their favorite foods (7–10), for meal ordering service in restaurants, it is also very important to understand what drives consumers' food choices and give recommendations through computational analysis of the variables collected both historically and at the tableside. Personalized recommendation systems using information and communication technologies (ICT) have been reported (11, 12). At present, these systems mainly satisfy specific needs expressed by consumers (13), such as healthy diet (14), balanced nutrition (15), and food taste (16). With the recent developments of machine learning, artificial intelligence, and cloud computing technologies, the development of smart food recommender systems for the general customers has been reported. For instance, a cloud-based smart restaurant management system (17) can provide easy-to-use interfaces to its users for food menu recommendation. Using advanced algorithms and Amazon Web Services (AWS), not only consumers can easily find their favorite food, but also restaurants can improve service, productivity and profits. Therefore, developing an intelligent menu recommender engine is an important task with promising applications to the enormous food service industry.

Beyond the field of dietary recommendation, many systems have been developed to predict people's interests (18). Personalized recommender engines play an increasingly important role in helping people make selections from overwhelming numbers of choices. For example, online stores such as Amazon, Netflix, and Pandora can recommend books, digital products, and other commodities. There have also been considerable recommenders in the academic field for students to choose schools, majors and classes (19–21). Regardless applications, existing recommender systems can typically be classified into three categories (22): (1) content-based, (2) collaborative, and (3) hybrid systems. The first category makes recommendations by matching item features; the second category makes predictions by analyzing rating data; and the last category possesses both content-based and collaborative features. Among different recommendation algorithms, the collaborative filtering (CF) algorithm and its variants have been used most widely (23). The CF-based algorithms can be further divided into memory-based and model-based algorithms (22, 24). It has been reported that, using a hybrid content-based collaborative filtering (CCF), the recommendation performance can be improved (25, 26). Recently, there has been a new progress in using matrix-factorization (MF)-based methods with high performance and scalability (27). The earliest version of the MF approach was based on singular value decomposition (SVD) (28). Lately, MF-based methods employed customer rating data to extract features and train a recommender based on predicted user preferences (29). There have also been cross integrations of CF recommender systems with regularized MF which have appeared at the Netflix prize competition (30). CF and MF based methods, along with their variations, have found various industrial applications (31–37). Relative to other fields, restaurant menu recommenders are less developed but some works have been reported. A real-time system was developed to monitor dining activity by videos (38). The system recommends additional dishes when the customers finish the existing ones and want more. Tan et al. (13) utilized the radio frequency identification (RFID) technology to improve food service. Elahi et al. (39) used tags and latent factors to design an interactive food recommendation system. Shaikh et al. (40) described a mobile recommendation system using context and user-profile information.

Some dietary recommendation systems pay special attention to the needs of customers who are patients. A system was developed to recommend foods based on user's illness and demographic information (41). Achieving a balanced nutrition was the focus of the recommender established using consumers' dietary records (42). Similarly, a recipe recommendation system was developed to help customers achieve fitness goals (43).

There were other recommender studies focusing on ingredients. Freyne et al. (44) and Feng et al. (45) extracted ingredients, which were individually rated by users, from menus. Recommendations were produced by weighting each ingredient. He et al. (46) used tastes (sour, sweet, bitter, spicy, and salty) to compose a vector of flavors for each dish. Then, customers' food ordering records and the established flavor vectors were used to make recommendations.

Incorporating other content information from recipes, MF-based recommenders generally achieved better preference prediction for users. Forbes and Zhu (47) proposed an algorithm incorporating the ingredient information into the MF method and improved the recipe recommendation performance. Lin et al. (48) employed main ingredients, courses, cuisines, etc. to obtain a recommender model. Although these food recommendation systems enhanced prediction accuracy, they directly incorporated the content information into item vectors for matrix factorization without revealing the hidden associations among these factors. As a result, these systems can only exploit explicit information about users' preferences. In order to reveal associations of food components, consumers' needs, and other related factors to produce better recommendations, we present a multi-attribute relation matrix tri-factorization (MARMTF) technique in this work. We first represent heterogeneous information as multi-type relation matrices. In addition to including users' ordering record and ratings for the dishes, we construct a set of relationship matrices, which reflects ingredients, spicy level, and price and integrate it into the recommendation framework. The multi-variant matrices are then integrated by data fusion using an advanced MF algorithm (49).

This paper is organized as follows. We introduce the recommendation strategies and methods in section Recommendation Strategies and Methods, where attribute relations for the food recommendation system are described. In section Experimental Studies, our experimental studies are presented which employ the multi-attribute relation matrix tri-factorization (MARMTF) framework to produce menu recommendation. Then, the performance of our recommendation system is discussed in section Results and Discussion. Finally, we draw conclusions in section Conclusion.

## Recommendation strategies and methods

### Theoretical background

It has been proven that Matrix factorization (MF) is both accurate and scalable for recommendation systems (27, 29). In this framework, a user-rating matrix is initially filled with the input data representing the collected information. Let the numbers of users and the pieces of information be *n* and *d*, respectively. Let **R** be a relation matrix describing the usefulness of the information items to the users. Thus, *n*×*d*→ **R**. Normally, this matrix **R** is sparse. Next, the rating matrix is factorized into two low-rank factor matrices. Finally, we estimate the unknown entries using the inner products of the matrices and the entries with the highest values are used to produce recommendations.

During the MF process, the non-negativity matrix factorization (NMF) is critically important. NMF aims to find two non-negative matrix factors **U** and **V** from a non-negative matrix **X**, i.e.,

(1) $$\begin{array}{l} \text{X}=\text{U}{\text{V}}^{T} \end{array}$$

where $\text{X}\in {\mathbb{R}}_{+}^{n\times d},\text{U}\in {\mathbb{R}}_{+}^{n\times c}$ and $\text{V}\in {\mathbb{R}}_{+}^{d\times c}$ [${\mathbb{R}}_{+}^{d\times c}$are all *d*-by-*c* matrices whose entries are non-negative. The rank *c* usually satisfies*c* ≪ min(*n, d*)].

Ding et al. (50) provided a systematic analysis of the NMF. It was shown that the NMF performs spectral clustering and the orthogonal NMF is equivalent to K-means clustering. Furthermore, Ding et al. (51) proposed a bi-orthogonal 3-factor NMF:

(2) $$\begin{array}{l} \underset{\text{F}\geq 0,\text{S}\geq 0,\text{G}\geq 0}{\min}\|\text{X}-\text{U}\text{B}{\text{V}}^{T}{\|}^{2},s.t.,\text{U}{\text{U}}^{T}=\text{I}\begin{array}{c} , \end{array}{\text{ V}}^{T}\text{V}=\text{I} \end{array}$$

where $\text{X}\in {\mathbb{R}}_{+}^{n\times d},\text{U}\in {\mathbb{R}}_{+}^{n\times k},\text{B}\in {\mathbb{R}}_{+}^{k\times l}$ and $\text{V}\in {\mathbb{R}}_{+}^{d\times l}$. Equation (2) can be called orthogonal non-negative matrix tri-factorization (ONMTF) which has a better capability in simultaneously clustering rows and columns of the data matrix. As an effective co-clustering tool, ONMTF was applied to collaborative filtering with improved performance (52).

Wang et al. (53) presented a novel symmetric penalized matrix tri-factorization (tri-PMF) framework which employs penalized terms for dyadic constrained co-clustering.

(3) $$\begin{array}{l} \underset{{\text{G}}_{1}\geq 0,{\text{G}}_{2}\geq 0}{\min}\|{\text{R}}_{12}-{\text{V}}_{1}{\text{B}\text{V}}_{2}^{T}{\|}^{2}+P\left(\chi_{1}\right)+P\left(\chi_{2}\right) \end{array}$$

where V_1_ and V_2_ denote the cluster indicator matrices of χ_1_ and χ_2_, respectively, and *P*(χ_1_) and *P*(χ_2_) correspond to the penalties on χ_1_ and χ_2_. Here the tri-PMF is extended to symmetric penalized matrix tri-factorization in order to cluster multi-type data objects simultaneously. Wang et al. (54) also proposed a symmetric non-negative matrix tri-factorization (S-NMTF) method to co-cluster multiple types of relational data.

### Multi-attribute relation fusion for food recommendation

Besides the user-dish rating data, our dish recommendation system also combines other relational data including dish-ingredients, dish-spices, and dish-price. Additional food-choice related factors, such as consumer's age, physical/medical condition, native region, meal time, season of the year, etc., may also be included. Based on the matrix tri-factorization techniques, we present a multi-attribute relational information fusion scheme which integrates available data sources to predict consumers' preferences.

#### Factorization model

In our recommendation model, the input data are relation matrices. If the *i*-th and *j*-th object types constitute a relation matrix **R**_*ij*_, then all relation matrices can be integrated to a block matrix **R**, given by

(4) $$\begin{array}{l} \text{R}=\left[\begin{array}{cccc} {}* & {\text{R}}_{12} & \cdots & {\text{R}}_{1r}\\ {\text{R}}_{21} & * & \cdots & {\text{R}}_{2r}\\ \vdots & \vdots & \ddots & \vdots \\ {\text{R}}_{r1} & {\text{R}}_{r2} & \cdots & * \end{array}\right] \end{array}$$

where the relation matrices between the same type of objects are denoted by the asterisk (“^*^”). For our recommendation system, if some relation matrices, such as user-ingredient, and ingredient-price, are not directly modeled, we let the corresponding locations be blank. Obviously, the relation matrices may not be symmetric, i.e., ${\text{R}}_{ij}\neq {\text{R}}_{ji}^{T}$.

Let us consider constraints of the relation between the same types of objects. Suppose that there are *r* data sources represented by a set of constraint matrices **P**_*i*_ for *i* ∈ {1, 2, ⋯ , *r*}. Constraints are collectively encoded in a set of constraint block diagonal matrices **P**

(5) $$\begin{array}{l} \text{P}=Diag\left({\text{P}}_{1},{\text{P}}_{2},\cdots \;,{\text{P}}_{r}\right) \end{array}$$

where *Diag*(·) denote the diagonalization for the block diagonal matrix **P**. In order to use all modeled relation matrices to obtain a fused block matrix, we first use matrix tri-factorization to decompose the original block matrix **R** into integrant block matrix factors **V** and **B**:

(6) $$\begin{array}{l} \text{V}=Diag\left({\text{V}}_{1}^{n_{1}\times k_{1}},{\text{V}}_{2}^{n_{2}\times k_{2}},\cdots \;,{\text{V}}_{r}^{n_{r}\times k_{r}}\right) \end{array}$$

(7) $$\begin{array}{l} \text{B}=\left[\begin{array}{cccc} {}* & {\text{B}}_{12}^{k_{1}\times k_{2}} & \cdots & {\text{B}}_{1r}^{k_{1}\times k_{r}}\\ {\text{B}}_{21}^{k_{2}\times k_{1}} & * & \cdots & {\text{B}}_{2r}^{k_{2}\times k_{r}}\\ \vdots & \vdots & \ddots & \vdots \\ {\text{B}}_{r1}^{k_{r}\times k_{1}} & {\text{B}}_{r2}^{k_{r}\times k_{2}} & \cdots & * \end{array}\right] \end{array}$$

Block matrix **B** has the same structure as **R** in Equation (4). From Equations (6, 7), we can reconstruct the block structure as **VBV**^*T*^:

(8) $$VBV^{T}=\left[\begin{array}{cccc} {}* & V_{1}B_{12}V_{2}^{T} & \cdots & V_{1}B_{1r}V_{r}^{T}\\ V_{2}B_{21}V_{1}^{T} & * & \cdots & V_{2}B_{2r}V_{r}^{T}\\ \vdots & \vdots & \ddots & \vdots \\ V_{r}B_{r1}V_{1}^{T} & V_{r}B_{r2}V_{2}^{T} & \cdots & * \end{array}\right]$$

#### Objective function and data processing procedure

The objective function aims at the closest approximation of the input data by the following minimization:

(9) $$\begin{array}{l} \underset{V\geq 0}{\min}J\left(\text{V};\text{B}\right)=\underset{{\text{R}}_{ij}\in ℜ}{\sum }\|{\text{R}}_{ij}-{\text{V}}_{i}{\text{B}}_{ij}{\text{V}}_{j}^{T}{\|}^{2}+tr\left({\text{V}}^{T}\text{P}\text{V}\right) \end{array}$$

where ||·|| and *tr*(·) denote the Frobenius norm and trace, respectively, and ℜ is the set of all relations included in our model. We can compute the factorization to obtain the latent factors **V** and **B** by solving the minimization problem with Equation (9). The factorization algorithm can be simply described as follows. Firstly, the matrix factors **V** and **B** are initialized (section Initialization of Decomposition Factors). Next, alternating between fixing **V** and updating **B**, and then fixing **B** and updating **V**, until the results achieve convergence to iteratively refine the latent matric factors. The update functions of **B** and **V** can be derived by multiplicative updating rules (49). For convergence criterion, run for a fixed number of iterations (section The Number of Iterations) is adopted in this study. Finally, we can use the convergent **B** and **V** to compute the approximation of input block matrix **VBV**^*T*^.

**Figure 1:**
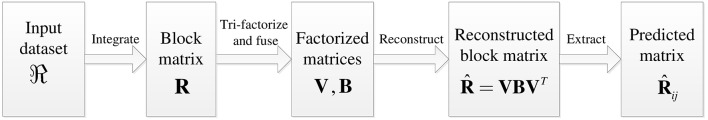
Flowchart of the recommendation system.

To predict users' preference ratings of different dishes, we reconstruct the rating matrix from the observed relation matrices. The whole processing procedure can be represented in Figure 1. In the step of matrix tri-factorization and fusion, we can use the multi-type relation matrices to obtain matrix factors **V** and **B**. Finally, the predicted rating matrix ${\hat{\text{R}}}_{ij}$ can be extracted from the reconstructed block matrix $\hat{\text{R}}$.

After the new user-dish rating matrix is generated, each dish is assigned with a predicted value. Then, the system will make the recommendation for users according to the ranking of dishes with adequate scores (determined empirically).

## Experimental studies

### Materials and datasets

Our experimental study was performed using Chinese foods which are renowned for their wide choices and varieties. First, we generated a list of Chinese foods commonly found in Chongqing, a major mid-west city of over 10 million, well-known for its spicy Sichuan cooking style. The foods selected were mostly in the low or moderate price range. Therefore, they have a large customer base. We recruited 37 adult evaluators (22 males and 15 females) who were all ethnic Chinese but were from different regions in China, not limited in Chongqing. They were healthy (based on their own evaluation), and their ages were between 20 and 60, for a better generalizability of our study. Each evaluator was presented with a list of 289 foods (dishes). He/she gave a rating for each dish according to his/her preference. The rating grades were integers within the range of 1–5, representing “hate,” “dislike,” “neutral,” “like,” and “love,” respectively. If the evaluator has no experience about a particular dish or was not sure because of a poor memory recall or other reasons, he/she simply left a blank for the dish. After all lists were collected, we integrate them to form the initial user-dish rating matrix exemplified in Table 1. For compactness of the table, we represent each dish with a sequential number. It can be seen that the matrix is, as it is normally, quite sparse.

**Table 1:** The user-dish rating matrix.

	***Dish*1**	***Dish*****2**		•••			***Dishn***		
*User*1								2	
*User*2					5				
			5	5					
		4				4			
•	5						3		
•						1			
•		4						3	
							2		
		2							
*User*m				3					4

As stated previously, food ingredients represent an important attribute for dishes. It is also one of the key factors driving consumers to choose their preferred dishes (55, 56). Therefore, we incorporated the ingredient-dish information, as exemplified in Table 2 where the dishes were classified into six main food ingredients: meat, poultry, vegetables, aquatic products, soybean products, and cereals. We used Boolean values to indicate whether a dish contains the particular ingredient (“1”) or not (“0”).

**Table 2:** The relationship matrix between the dishes and the food ingredients.

	**Meat**	**Poultry**	**Vegetables**	**Soybean products**	**Cereals**	**Aquatic products**
*Dish*1	1	0	0	0	0	0
*Dish*2	0	0	0	0	1	0
	0	1	1	0	0	0
	0	0	0	1	0	0
•	1	0	0	1	0	0
•	0	0	1	0	0	1
•	0	0	0	1	0	1
	1	0	0	0	0	0
	1	0	0	0	1	0
*Dish*n	0	0	1	0	0	0

For the Sichuan and many other Chinese cuisine systems, the degree of spiciness is important for people to make food choices (57). Some studies have been conducted on the factors that influence consumers' behavior of eating spicy food (58–60). Therefore, we also utilized spiciness as an important factor for consumers' food choices. The spicy level of each dish is commonly available in restaurants' menus (e.g., indicated by the number of hot pepper symbols). Using this information, the dishes were classified in four levels: “not spicy,” “slightly spicy,” “medium spicy,” and “very spicy.” Table 3 describes the relationship matrix of the dishes and their spiciness.

**Table 3:** Relationship matrix of the dishes and their spicy levels.

	**No spicy**	**Slightly spicy**	**Medium spicy**	**Very spicy**
*Dish*1	0	0	0	1
*Dish*2	0	0	1	0
	0	1	0	0
	0	1	0	1
•	0	0	1	1
•	0	1	0	0
•	0	0	0	1
	1	0	0	0
	1	0	0	0
*Dish*n	0	0	1	0

Additionally, price is an undeniable factor influencing food choices, especially for low- and middle-income consumers (61, 62). For people dining away from home, food consumption is largely responsive to price change (63). Therefore, we incorporated food price into our recommendation system, as shown the dish-price relation matrix in Table 4 where three price levels were extracted from the restaurant menu: “low price,” “medium price,” and “high price.”

**Table 4:** Relationship matrix between the dishes and the price levels.

	**Low price**	**Medium price**	**High price**
*Dish*1	1	0	0
*Dish*2	0	1	0
	0	1	0
	1	0	0
•	0	0	1
•	1	0	0
•	0	0	1
	0	1	0
	0	1	0
*Dish*n	0	0	1

### Relation integration

**Figure 2:**
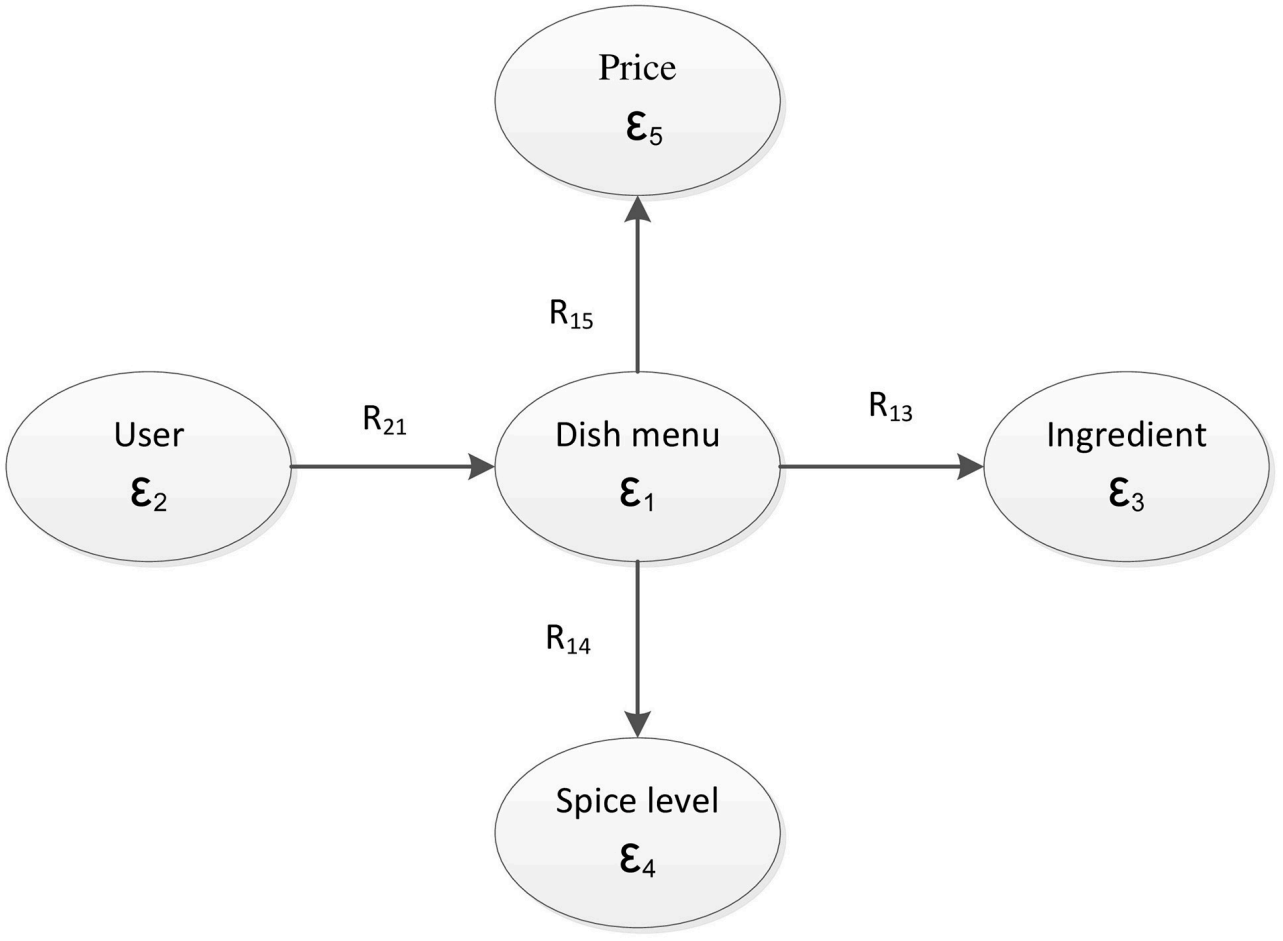
Relation fusion graph of the system.

In terms of mathematical modeling, we have formed 5 object types, ε_1_ through ε_5_, corresponding to “dish,” “user,” “food ingredient,” “price level,” and “spicy level.” For convenience in implementation, each relationship matrix was represented in the Comma Separated Value (CSV) format. From these data sources, we integrated them into a relation graph as shown in Figure 2. The attribute relations of user-dish, dish-ingredient, dish-price, and dish-spicy were represented by **R**_21_, **R**_13_, **R**_14_, and **R**_15_, respectively. We used these relation matrices as input data for the matrix tri-factorization model.

### Data processing and analysis

We collected input data from 37 evaluators for 289 dishes (described in section Materials and Datasets), along with the preparation of the relation matrices described above. The user-dish rating dataset was then divided into two sets, training set (83.3% of data) and test set (16.7% of data).

#### Evaluation metrics

The root mean-squared error (RMSE) and mean absolute error (MAE) were utilized to evaluate the performance of our recommendation systems (18), given by

(10) $$\begin{array}{l} RMSE=\sqrt{\frac{1}{\left|T\right|}\underset{\left(u,i\right)\in T}{\sum }{\left(r_{ui}-{\hat{r}}_{ui}\right)}^{2}} \end{array}$$

(11) $$\begin{array}{l} MAE=\frac{1}{\left|T\right|}\underset{\left(u,i\right)\in T}{\sum }|r_{ui}-{\hat{r}}_{ui}| \end{array}$$

where *r*_*ui*_ and ${\hat{r}}_{ui}$ denote the ratings given by the user *u* and the recommendation system for item *i*, respectively, and |*T*| denotes the number of elements in rating set *T*.

#### Initialization of decomposition factors

The initialization of factor matrix **V** in Equation (1) is important because system performance is sensitive to **V**. The initialization also influences the convergence of the algorithm. We adopt the random Acol method (64) to initialize **V** in which the initialization of each column of **V** is formed by averaging random columns of **R**. Our algorithm derives factors **B** in Equation (2) from **V**, as described in section Objective Function and Data Processing Procedure. In addition to the initialization, there are two important parameters, the factorization rank and the number of iterations, to be discussed below.

#### Factorization rank

**Figure 3:**
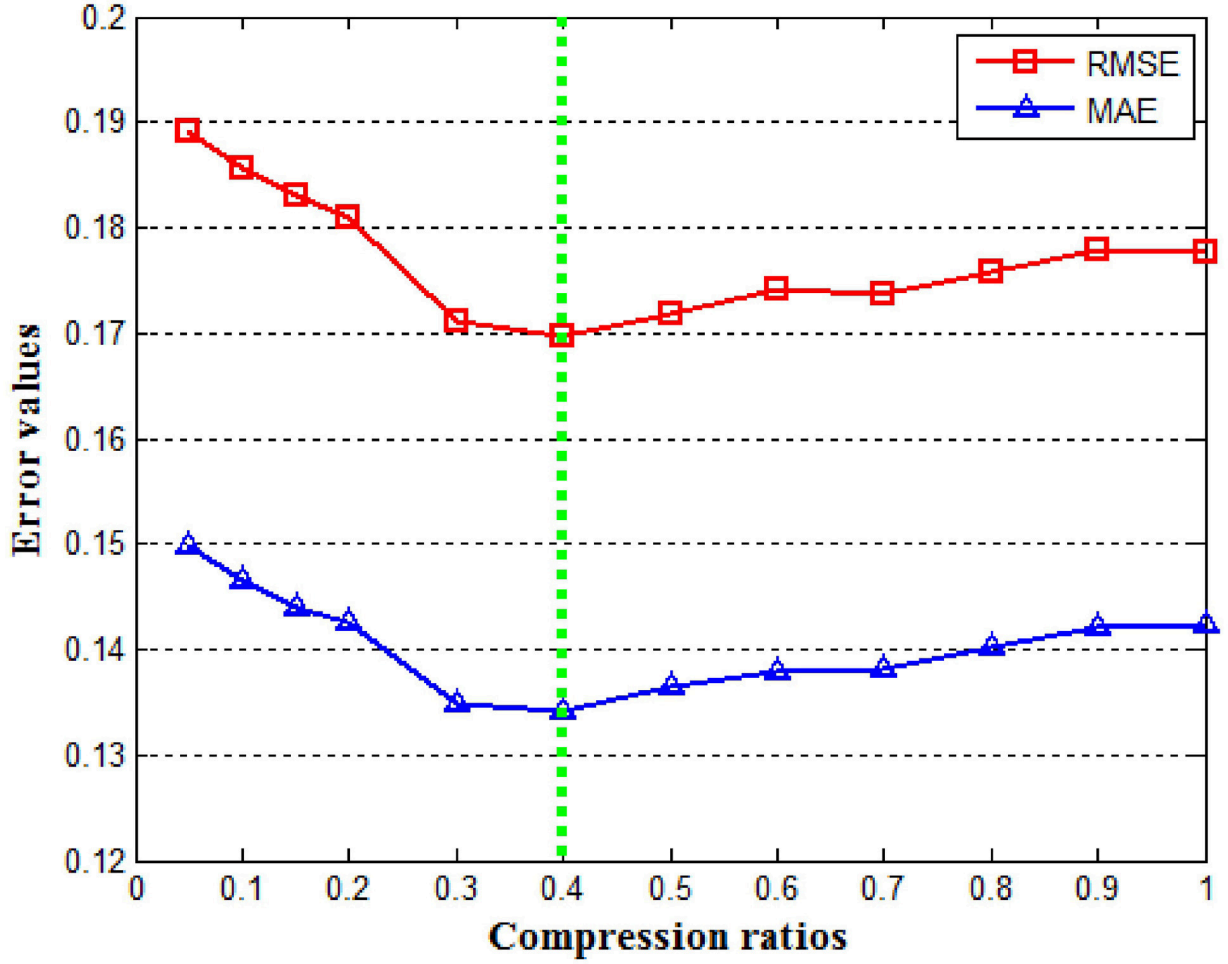
Effect of the different factorization ranks. Both the RMSE and MAE are minimized at a compression ratio near 0.4.

In matrix tri-factorization formula ${\text{R}}_{ij}-{\text{V}}_{i}{\text{B}}_{ij}{\text{V}}_{j}^{T}$, the dimensions of ${\text{R}}_{ij}\in {\mathbb{R}}^{n_{i}\times n_{j}}$ and ${\text{B}}_{ij}\in {\mathbb{R}}^{k_{i}\times k_{j}}$ are *n*_*i*_ × *n*_*j*_ and *k*_*i*_ × *k*_*j*_, respectively. *k*_*i*_ and *k*_*j*_ are factorization ranks which are smaller than *n*_*i*_ and *n*_*j*_. Therefore, the matrix factor **B**_*ij*_ can be considered as a compressed version of the original matrix **R**_*ij*_ (65). The factorization ranks determine the degree of dimension reduction for the object types. In our study, we use the dimension compression ratios kini and kjnj to denote the degree of dimension reduction determined by the selected factorization ranks *k*_*i*_ and *k*_*j*_. The ratios affect the performance of our data fusion model. For each ratio, if it is too large, the clustering becomes overly fine. On the other hand, if it is too small, the clustering tends to be rough. In order to simplify parameter tuning, we let kini=kjnj for all *i* and *j*. To find the dimension compression ratio that optimizes the quality of the system, we fixed the number of iterations at 200, varied the unified compression ratio between 0 and 1, and utilized the RMSE and MAE defined in (10) and (11) to measure performance. Our result is shown in Figure 3. It can be observed that the optimal value of the compression ratio is ~0.4 which was selected.

#### The number of iterations

**Figure 4:**
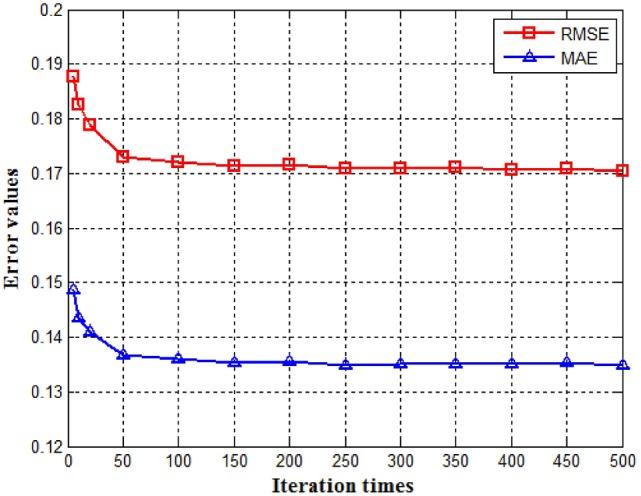
RMSE (top curve) and MAE (bottom curve) vs. the number of iterations.

The objective function *J*(**V;B**) given by Equation (9) can be minimized by multiplicative updating for **V** and **B**. Since it is an iterative process, the number of iterations must be determined. We determined it experimentally by observing the convergence of our system. It can be seen from Figure 4 that both RMSE and MAE decrease as the number of iterations increases. However, when it reaches 100, the error reduction becomes insignificant. We therefore selected the number of iterations to be 200 with a sufficient safety margin.

We implemented our 3-factor matrix factorization algorithm (unoptimized) in Python 3.5 edition on a laptop with an i5 core. The execution time was ~15 s. Despite the relative slowness in this implementation, we believe that the computational efficiency can be reduced significantly by optimizing the algorithm and utilizing a parallel processor, such as a GPU.

## Results and discussion

Using the optimally determined parameters, we constructed our recommendation system using the training set, which was composed of 83.3% of the total data. Once constructed, we utilized the test set, composed of the rest of collected data, to evaluate performance based on the RMSE and MAE metrics. For reliability of the output, the test procedure was repeated 10 times, and we took the average of the evaluation results.

**Figure 5:**
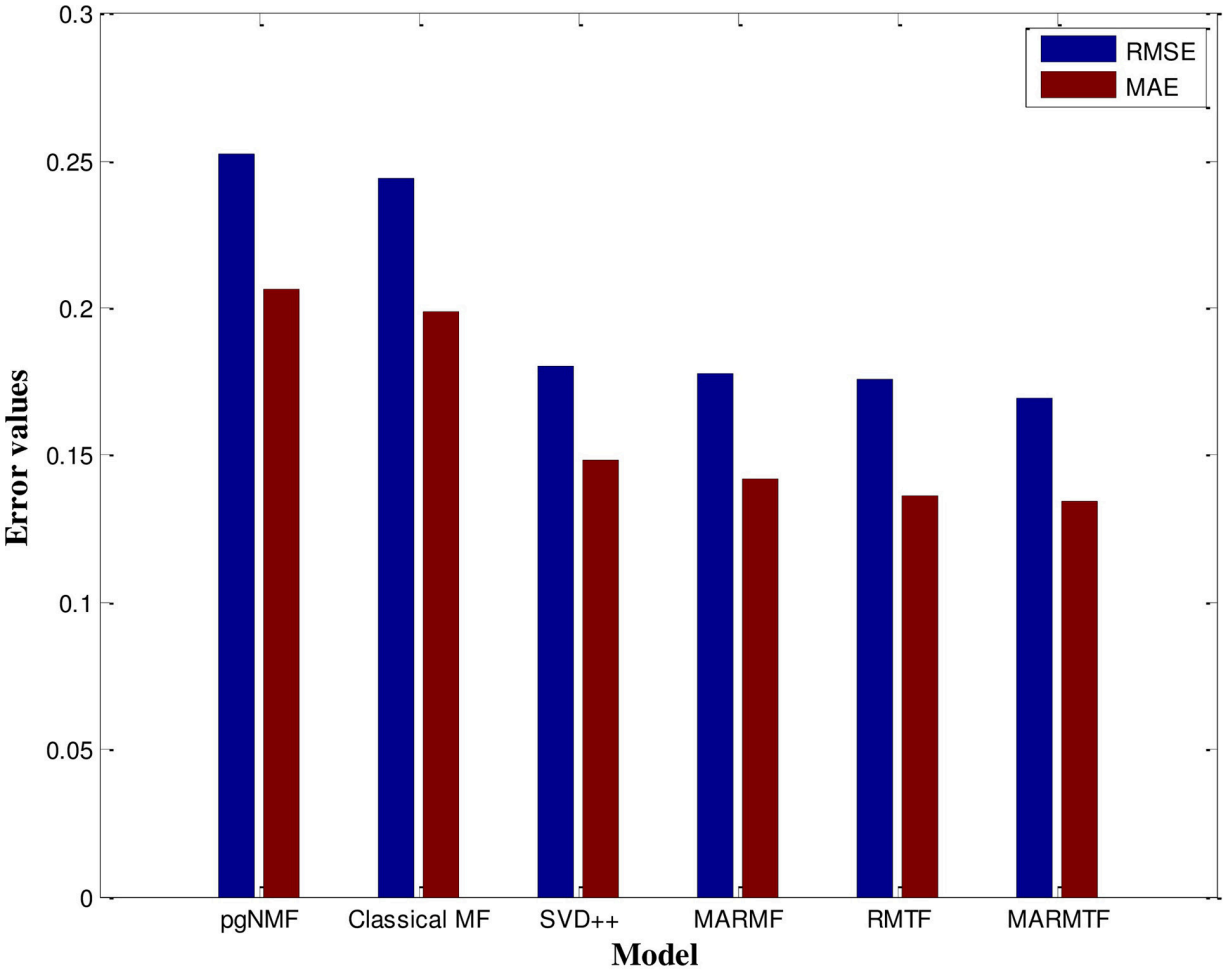
RMSE and MAE for different models.

Additionally, we compared our method with several commonly used methods, including projected gradient NMF (pgNMF) (66), classical matrix factorization (MF) (29), and SVD++ (67). Furthermore, we compared our current use of 3-factor matrix factorization (MARMTF) with an alternative of using 2-factor matrix factorization based on the same multi-type relation dataset. Finally, in order to validate our approach of using multi-type relation data, we performed the matrix tri-factorization using only the user-dish rating matrix. The comparison results can be observed in Figure 5. The MARMTF model achieve the best prediction rating accuracy.

For more details, our results are listed in Table 5. It can be observed that the RMSE and MAE have the minimum values at 0.1693 and 0.1342, respectively, indicating that the MARMTF method outperformed other algorithms. The next performers are the rating matrix tri-factorization (RMTF) and multi-attribute relation matrix factorization (MARMF) methods which are better than the traditional methods, including SVD++, classical MF, and pgNMF.

**Table 5:** Accuracy comparison of recommenders.

**Model**	**RMSE**	**MAE**
pgNMF	0.2527	0.2067
Classical MF	0.2443	0.1989
SVD++	0.1805	0.1482
MARMF	0.1776	0.1422
RMTF	0.1757	0.1365
MARMTF	0.1693	0.1342

In this comparison, the recommendation models utilize different amounts of information and/or treated the information differently. For example, the projected gradient NMF model assumes that an absence of rating implies an unfavored item, and the classical MF model only utilizes the rating matrix (68). In contrast, the SVD++ is a matrix factorization model that can combine mean rating, user-item bias, and implicit feedback information (69). As a result, the prediction accuracies of the SVD++ and other multi-attribute methods are higher than those of the projected gradient NMF and Classical MF. Thus, our results agree with a previous report that a recommendation model generally achieves a better performance if it incorporates more background information (67). However, it is difficult for the existing techniques to fuse a large number of attributes from a wide variety of resources.

With regard to making dish recommendations for consumers in restaurants, the relation matrices must be constructed with multiple attributes. Therefore, it is important to integrate and fuse the information from different sources. Our MARMTF model decomposes all the relation matrices systematically for the reconstruction of the rating prediction matrix. In addition, this model achieves a better clustering accuracy by simultaneously co-clustering multiple attributes simultaneously. Due to these valuable properties, the prediction accuracy of the MARMTF overperforms the MARMF which adopts 2-factor matrix decomposition. Overall, the MARMTF can better “understand” complex underlying relationships from different sources to produce more relevant recommendations for consumers.

Despite the advantages, we point out that the current version of the MARMTF has certain limitations. The evaluation was performed in a particular region (Chongqing) where foods tend to be spicy. As a result, there is a tendency that our food choice preferences are biased toward spicy foods and our results are subject to regional limitations. Additionally, we adopted only food ingredients, spicy levels, and price levels as the factors to help consumers choose food. Therefore, the attributes utilized are limited. In future studies, we plan to enhance our recommendation model by considering additional attributes, such as time of meal, season of the year, native region of the consumer, etc., under the MARMTF framework. Finally, food recommendations for healthy diet and balanced nutrition are of great interests for people with chronic diseases or being overweight. In order to produce health-awareness recommendations, we plan to use both food preference and demographic/medical data [e.g., age, body mass index (BMI), existing chronic conditions, etc.] and apply the MARMTF model to make dietary recommendations.

## Conclusion

With improvements in food production and services, consumers are facing with increased food products and diverse eating environments which make food-choice decisions more complex (70). In order to provide an effective meal selection tool for consumers in restaurants, we have developed a food recommendation system incorporating the information about eating behaviors and food attributes. A multi-attribute relation matrix tri-factorization framework has been presented. Based on the user-dish rating matrix, our relation-driven recommendation model utilizes other dish attribute relation matrices, including dish-ingredients, dish-spices, and dish-price, as the input data to predict consumers' food choices. Experimental results using real-world data have shown that the MARMTF model achieved better performance than existing recommendation methods. In the future work, we will incorporate more information and attributes, not only for choosing favorable food, but also for healthy eating and balanced nutrition.

## Author contributions

All of the authors contributed to the study conception and design, data collection, and interpretation of findings. XL conducted the statistical analyses and drafted the manuscript. MS performed the study design, interpretation of findings, and revised the manuscript. WJ advised on the statistical analysis and reviewed drafts of the manuscript.

### Conflict of interest statement

The authors declare that the research was conducted in the absence of any commercial or financial relationships that could be construed as a potential conflict of interest.
